# Foot Oligodactyly as the Main Dysplasia in Children

**DOI:** 10.7759/cureus.34896

**Published:** 2023-02-12

**Authors:** Nickolaos Laliotis, Panagiotis Konstantinidis, Chrysanthos Chrysanthou

**Affiliations:** 1 Orthopaedics, Interbalkan Medical Center, Thessaloniki, GRC; 2 Anatomy and Surgical Anatomy, Aristotle University of Thessaloniki, Thessaloniki, GRC

**Keywords:** oligodactyly, leg length discrepancy, absent foot rays, fibular hemimelia, foot dysplasia

## Abstract

Introduction

Foot oligodactyly is usually associated with fibular insufficiency or cleft foot syndrome. A foot with a reduced number of rays may occasionally have an isolated dysplasia.

Methods

We reviewed the clinical notes and X-rays of six children with oligodactyly, having a normal development of the tibia and fibula. Clinical evaluation recorded the plantigrade or deviated foot, appropriate shoe wear, and aesthetic presentation of barefoot children. Radiological examination revealed missing or hypoplastic bones in the foot, the presence of other deformities, and leg length discrepancy (LLD) of the affected limb.

Results

On clinical evaluation, all children except one had a plantigrade foot with normal shoe wear; the lesion was not spotted in three of them unless informed of the presence of the dysplasia. Radiological examination in four of them revealed the absence or hypoplasia of the navicular, with a normal shape of the first metatarsal. Calcaneocuboid joints were normal in five of them; LLD was the main problem in three children. The girl with bilateral oligodactyly presented as a normal child.

Conclusion

Oligodactyly may present as an isolated dysplasia. LLD in these patients, which is less severe than in children with fibular or tibial insufficiency, is the main issue that requires surgical management in later life. Prenatal diagnosis of oligodactyly as an isolated dysplasia is an important feature for appropriate counseling of parents.

## Introduction

Evaluation of the foot of a child with less than five rays requires thorough investigation. It can be part of a paraxial fibular or tibial deficiency or a cleft foot deformity. Fibular hemimelia is a longitudinal defect either terminal or intercalary and is associated with several deformities, including valgus knee, ball joint ankle, tarsal coalition, and more prominently severe leg length discrepancy (LLD), which is the main feature [1-3]. A cleft foot is a failure of formation usually of the central rays but in more severe forms it may present even as monodactyly. A web exists in the area of the absent rays. It is described with other malformations as cleft hand, syndactyly, or tibial deficiency. A cleft foot has been reported with a variety of syndromes (Cenani-Lenz, Ectrodactyly-ectodermal dysplasia-cleft syndrome) [4-9].

Foot oligodactyly may be found as isolated dysplasia with a normal fibula. There are absent rays usually affecting the central rays, associated with osseous dysplasia involving the bones of the foot. It was previously described as terminal hemimelia, with an intermediate ray deficiency. LLD is expected to be small, and with the development of the child, it may be the main problem that requires treatment [10,11]. With the evolution of prenatal diagnosis of oligodactyly, appropriate counseling is important for parents. We can inform them of the benign course in case of isolated oligodactyly, without elements of paraxial deficiency [12-14].

We present six children with oligodactyly, with normal tibia and fibula development, who were separated from another group of children with a reduced number of foot rays that are associated with fibular or tibial deficiency. In this study, we evaluated and discuss the clinical and radiological findings. There were various malformations of the bones in the medial and central rays. The course of the deformity was benign, with a moderate LLD in children having unilateral involvement.

## Materials and methods

We reviewed the clinical pictures and radiological examinations of a group of 11 children (15 feet) who had been under our care during the last 15 years with reduced rays of the foot. The age of the children at the initial examination ranged from one week to three years.

These children were analyzed and divided into two groups. The first group of six children had isolated dysplasia with the absence of foot rays, and normal development of the tibia and fibula of the affected limb. In the second group of five children, where foot dysplasia was part of the hemimelia, four of them had a fibular deficiency and one had a bilateral tibial deficiency. There were another three children with a five-ray foot in the group of children with fibular hemimelia. We further excluded another two children with oligodactyly and cleft foot, as they were in a different dysplasia group.

The group of six children with isolated foot oligodactyly was analyzed using clinical evaluation and appropriate radiological assessment. There was a two to seven years follow-up. On clinical examination, the foot was recorded as normal plantigrade or with deviation in varus, valgus, or equinus. The stability of the ankle joint was examined both clinically and radiologically with the parallel position of the talus in the ankle mortise. The ball joint ankle was reported based on radiographic findings. The types of footwear and participation in daily activities were also reviewed. Parents were asked whether dysplasia was noticed by others when their children were barefoot.

Radiological examinations were performed, including anteroposterior (AP) and lateral radiographs of the feet, AP and lateral radiographs of the tibiae, and radiological examination of the femora. The first X-rays were performed up to the age of six months and were repeated annually. LLD was assessed using digitalized X-rays.

This is a retrospective study, having the approval of the Interbalkan Medical Center ethical committee on 2/5-01-2023.

## Results

The initial evaluation was performed in the first two months of age in three of the children. They had a prenatal diagnosis of oligodactyly, and two of them underwent prenatal counseling with the senior surgeon. In a report by a referral gynecologist, apart from the absence of foot rays, measurements of the tibia and femur were normal and no other abnormalities were noted. We informed the parents that in case of foot deviation development, this can be managed and a follow-up for possible mild leg length discrepancy as the child grows is necessary.

In all six children, there was oligodactyly with four-ray feet, apart from one child that had three-ray feet. The proximal part of the limb, hip, femur, and tibia appeared normal with the presence of the fibula. Limb length discrepancy appeared from the initial clinical assessment in all children with unilateral oligodactyly.

**Figure 1:**
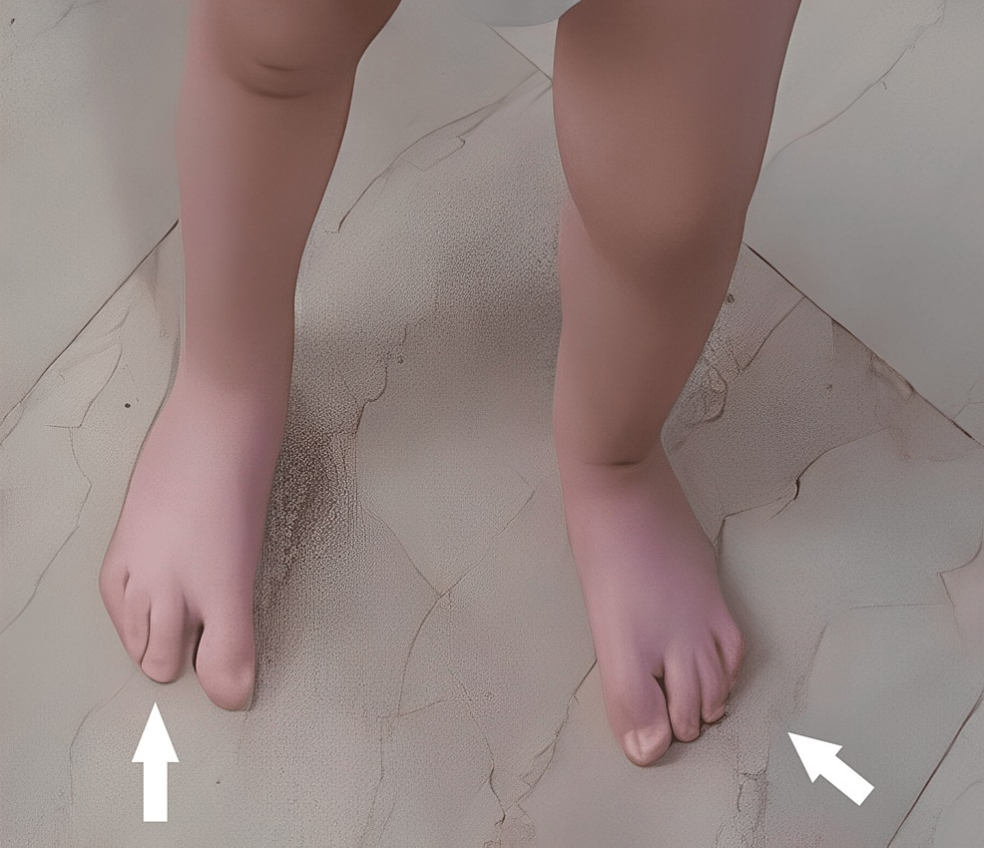
1st patient's feet at the age of 2 years.

**Figure 2:**
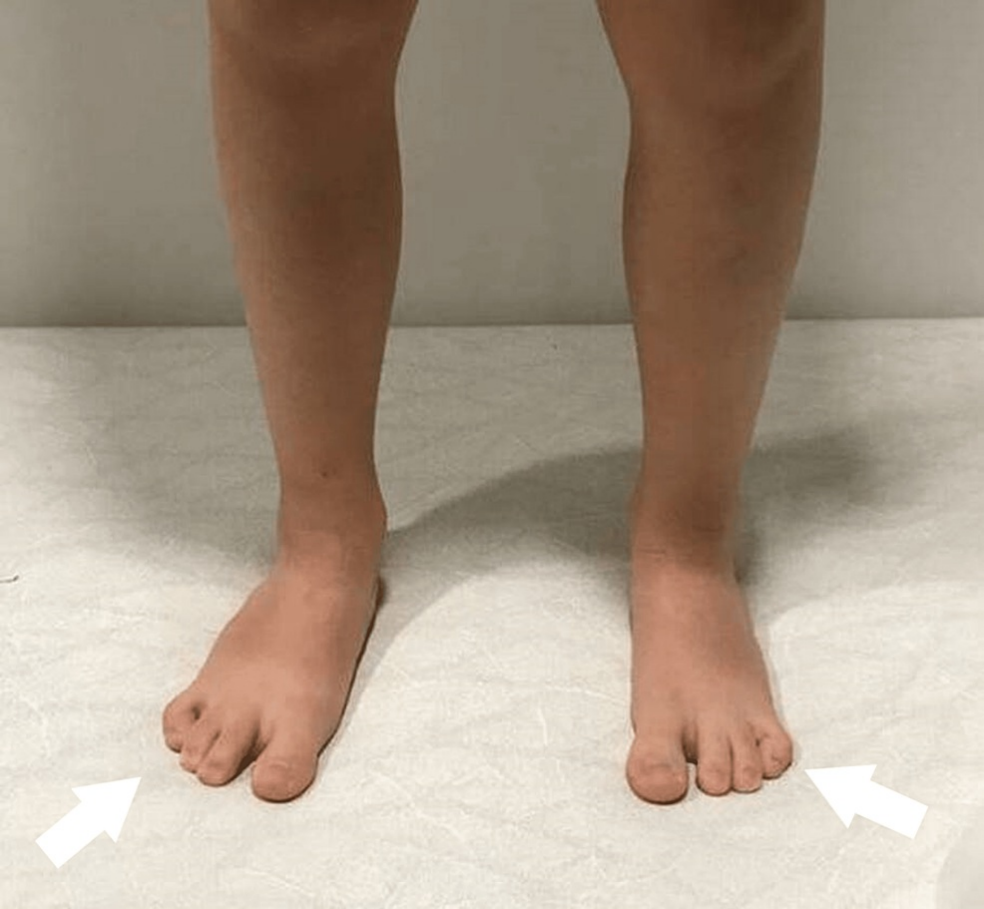
1st patient's feet at the age of 7 years - anterior view

**Figure 3:**
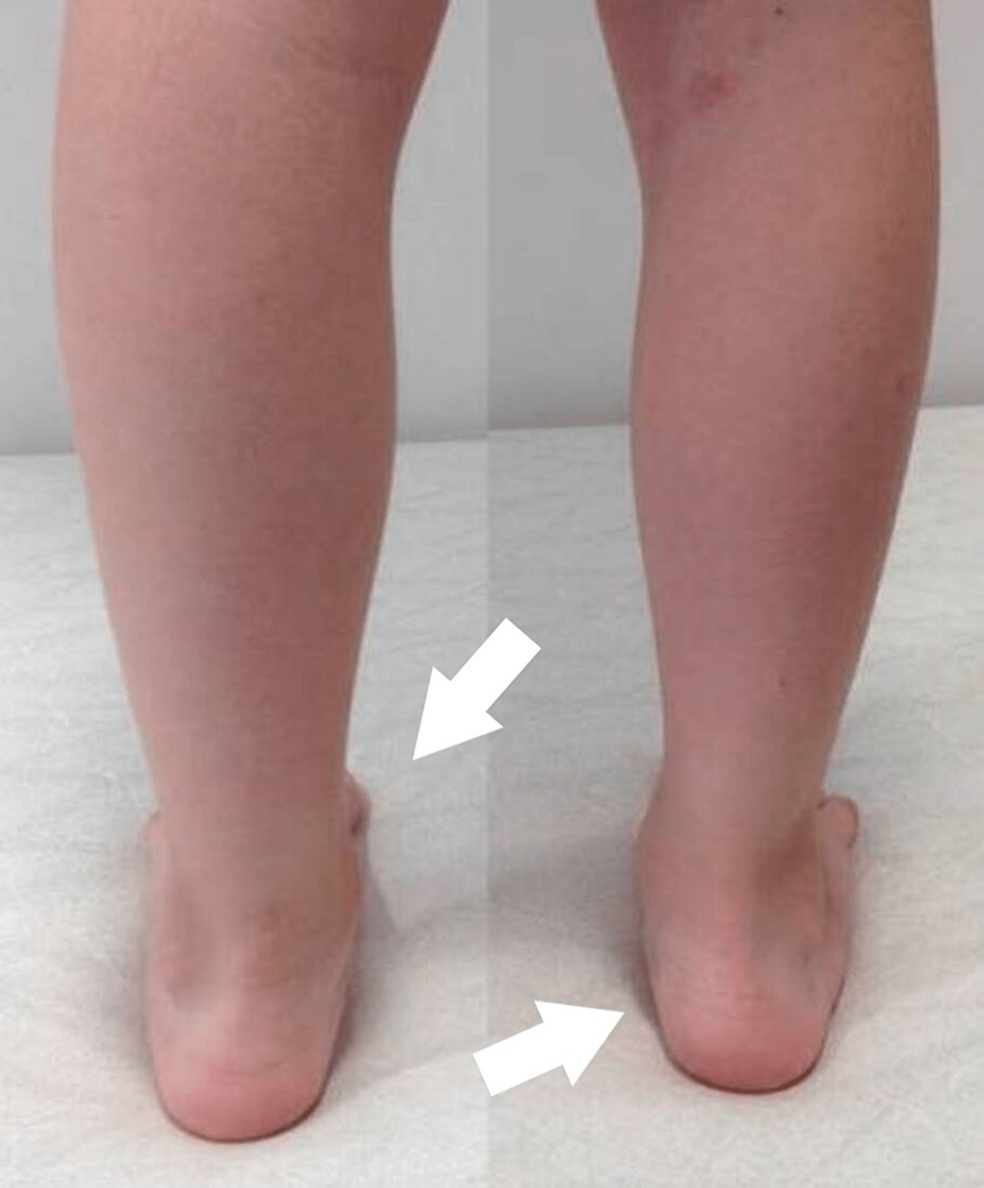
1st patient's feet at the age of 7 years - posterior view

In the female patient, the dysplasia appeared bilateral. The four-ray foot was unnoticed with bare feet, unless in purpose mentioned. The feet had a normal plantigrade shape and there was no obvious deformity of the feet. She has normal ankle and subtalar movements. She has a normal foot size, using the modern trend for shoes (Figures 1-3).

**Figure 4:**
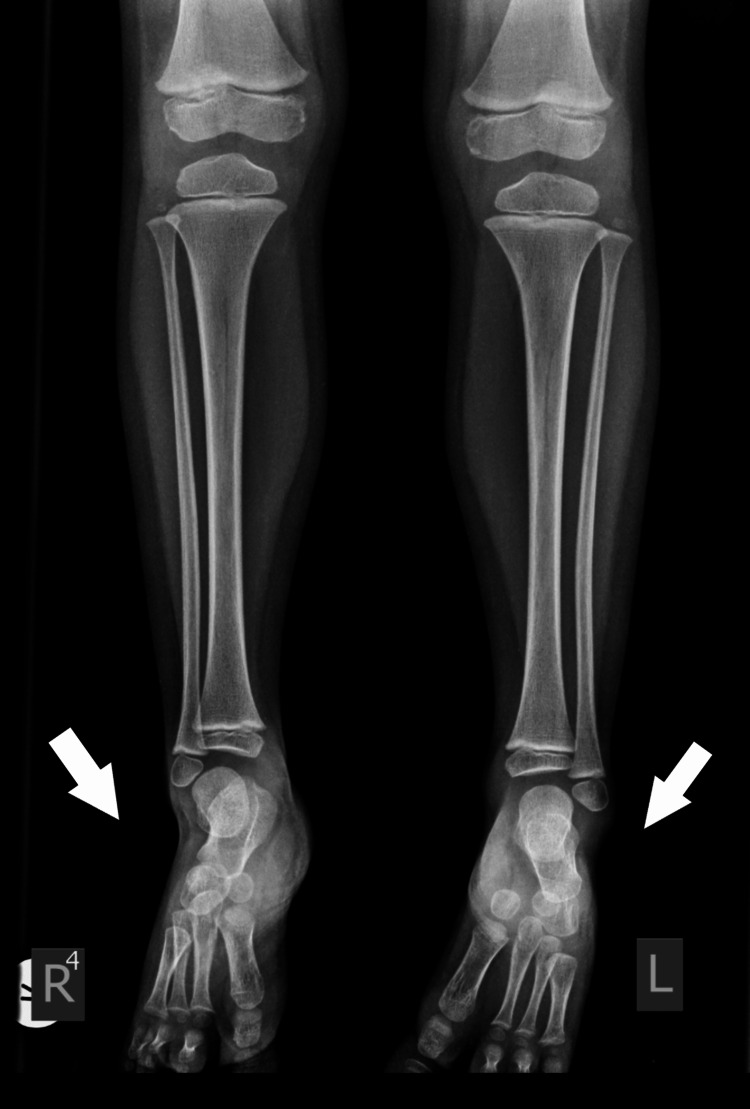
X-ray of 1st patient's feet with bilateral involvement at the age of 2 years. Normal development of the tibia and fibula. Delayed initial ossification of the navicular.

**Figure 5:**
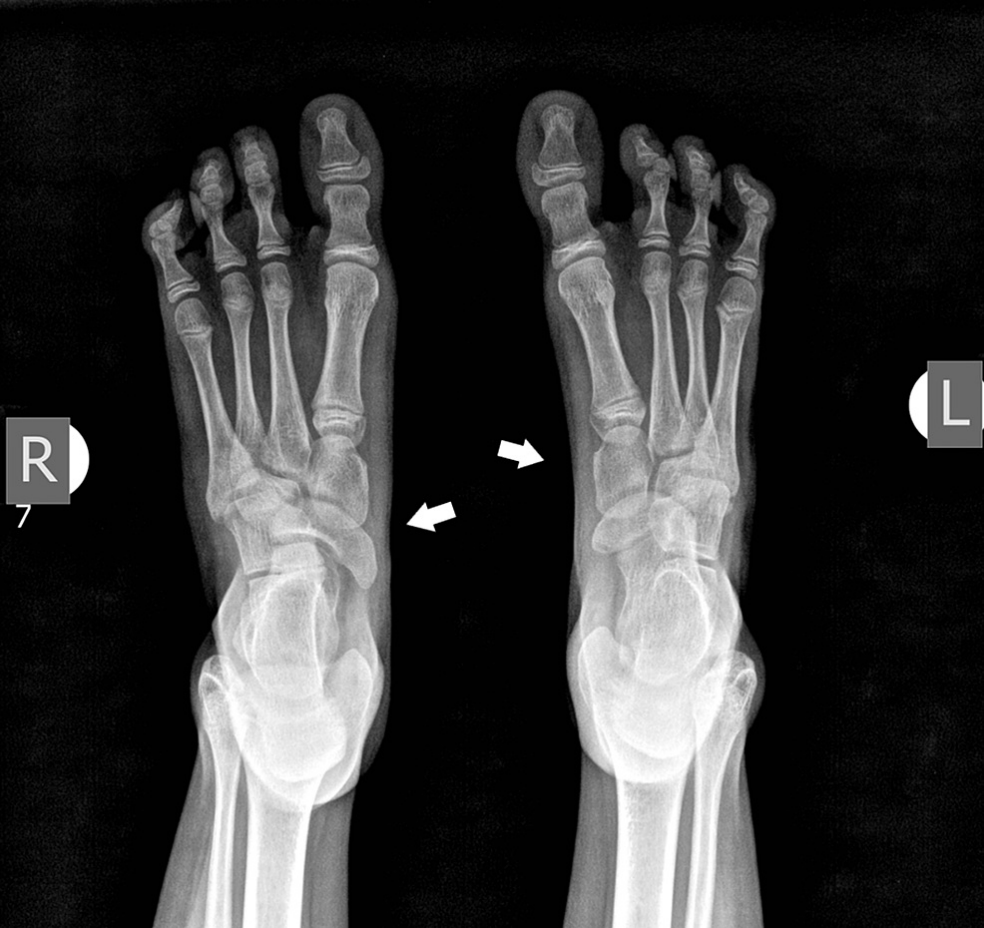
X-ray of 1st patient's feet at the age of 7 years with an enlarged navicular, with a coalition of the talus navicular, and normal bilateral calcaneocuboid joint. There are two cuneiforms present.

Plain X-rays showed normal development of the fibula and normal ankle joint. The calcaneo-cuboid joint was present bilaterally. The navicular had delayed ossification but appeared enlarged at the age of 7 years. There was a coalition with the talus, on the left side. There were two cuneiforms present and it was difficult to recognize which one was missing (Figures 4, 5).

**Figure 6:**
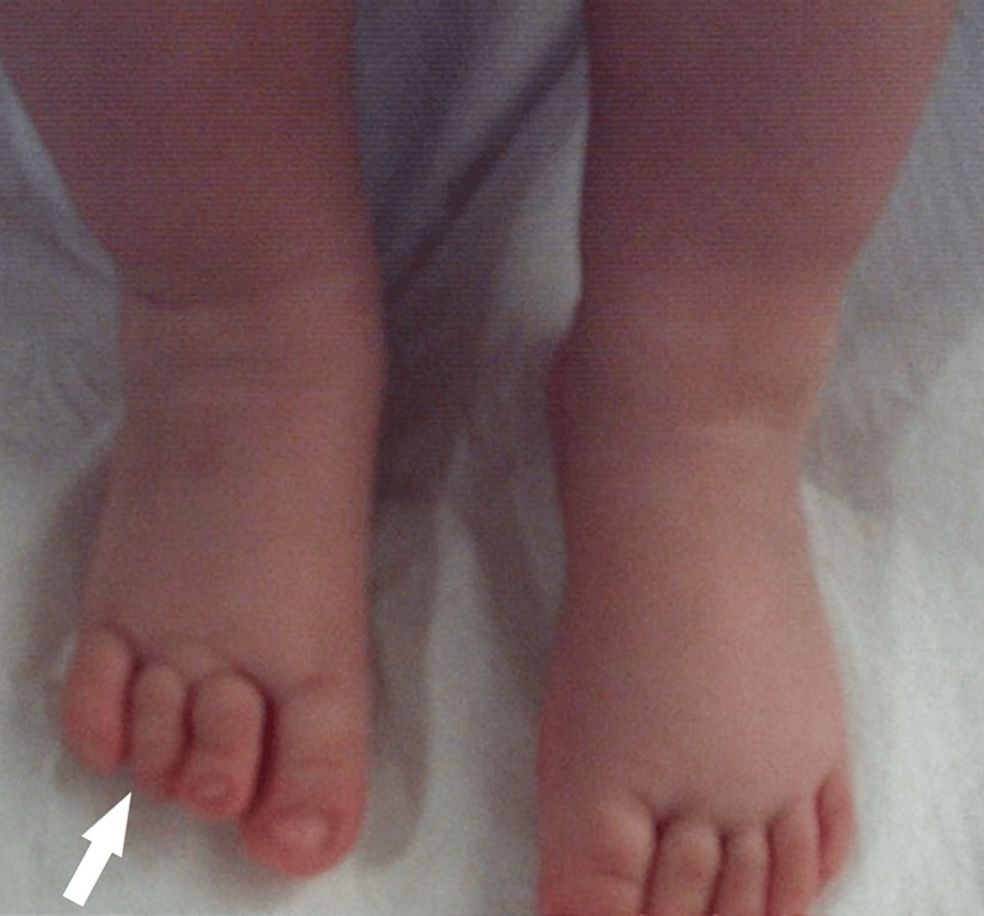
2nd patient's unilateral oligodactyly, with a marginally smaller foot, difficult to be spotted unless it was notified at the age of 1 year.

**Figure 7:**
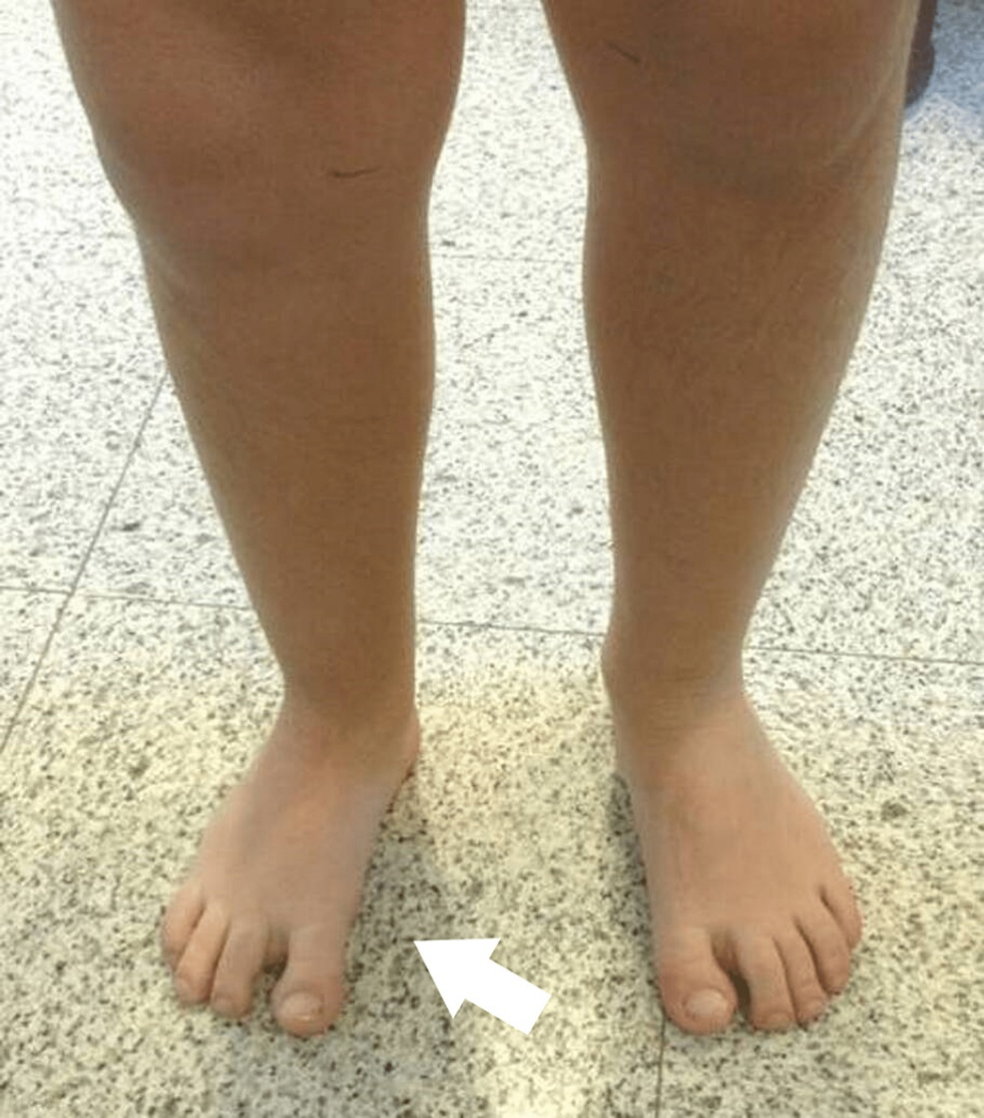
2nd patient's unilateral oligodactyly, with a marginally smaller foot, difficult to be spotted unless it was notified at the age of 5 years.

**Figure 8:**
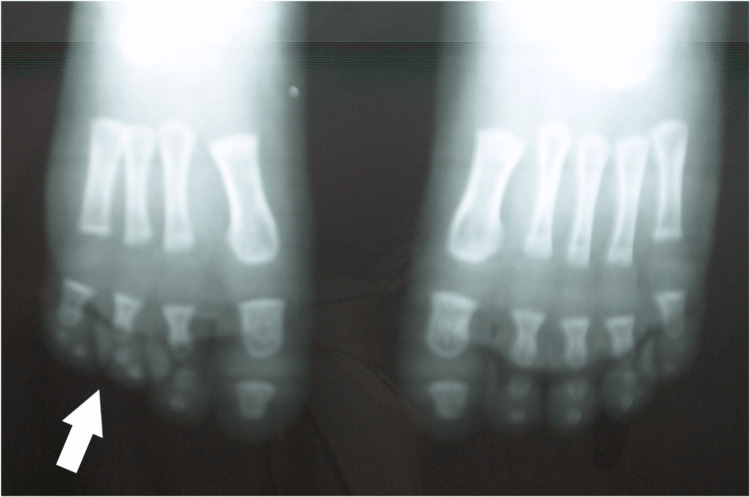
X-ray of the 2nd patient's feet, with a smaller length of the metatarsal at the age of 1 year.

**Figure 9:**
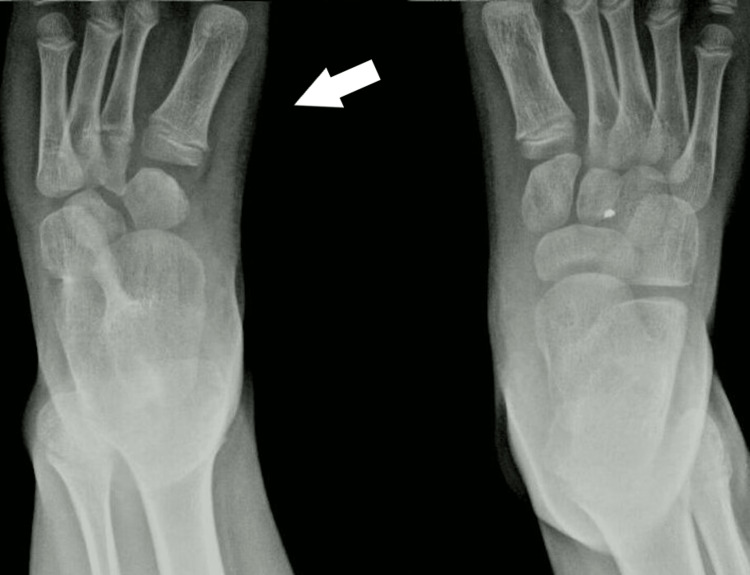
X-ray of the 2nd patient's feet, at the age of 5 years with a calcaneocuboid coalition of the right foot, two cuneiforms, and complete absence of the navicular.

The next four children had unilateral involvement with a four-ray foot. In two of them, it was difficult to spot the difference unless informed of the reduced rays when standing (Figures 6-9).

They all had an LLD with a ball joint ankle, without signs of instability.

**Figure 10:**
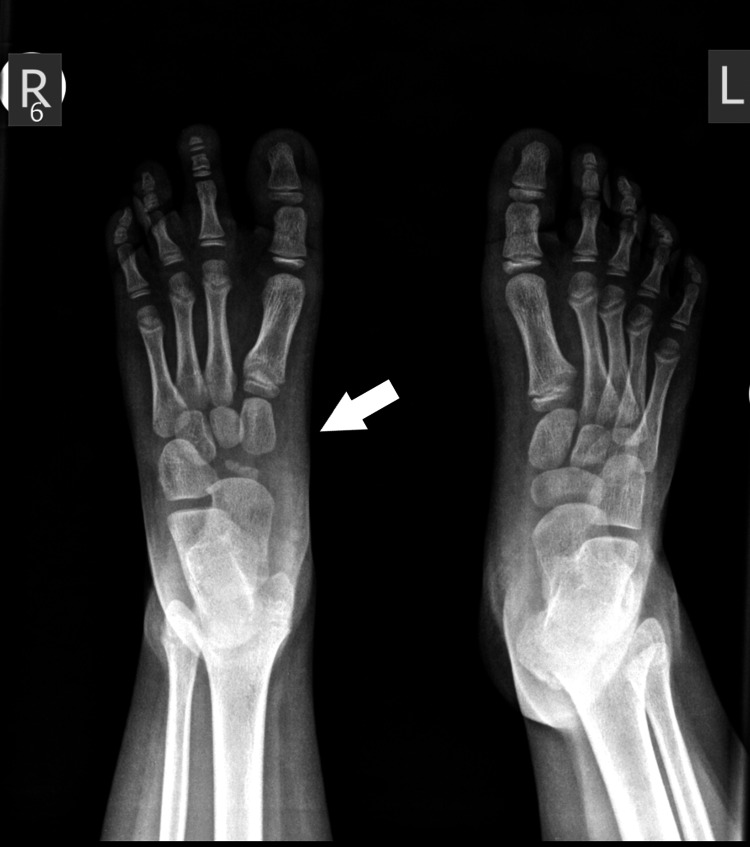
X-ray of the 3rd patient's feet, with normal calcaneal cuboid joint, hypoplastic navicular, and presence of three cuneiforms.

**Figure 11:**
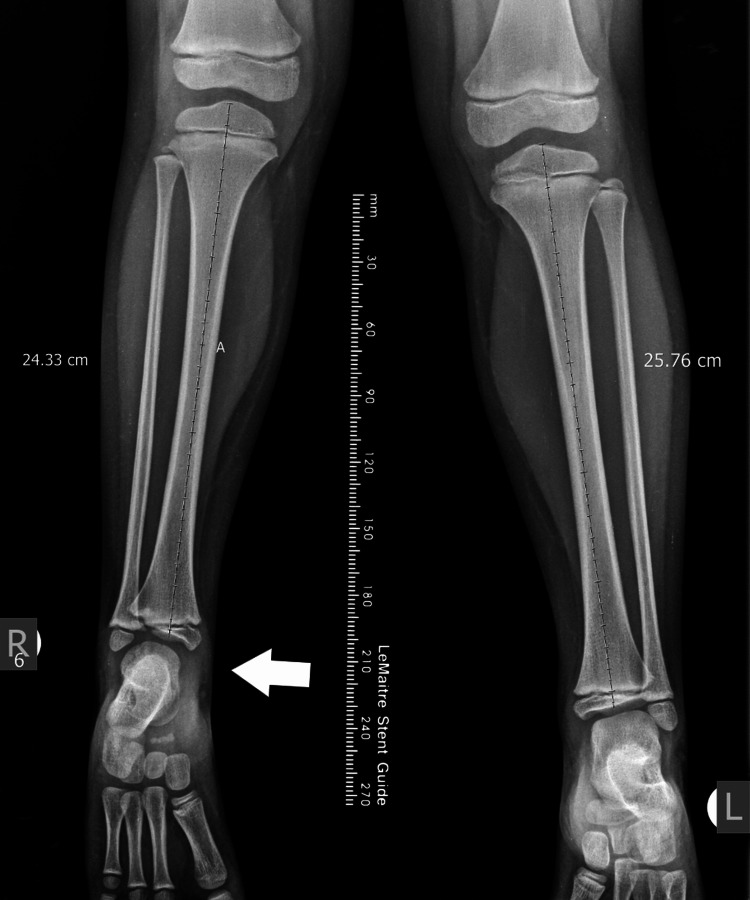
X-ray of the 3rd patient's feet with LLD 1.43 cm, and the ankle joint has a ball joint figure. LLD: Leg length discrepancy

Both had slightly smaller feet in length, apparent as well on X-ray films. The cuboid was present; in one case, there was a calcaneocuboid coalition. One child had three cuneiforms and the other had two cuneiforms. The navicular was hypoplastic in the first child and absent in the second. Normal development of the 1st metatarsal and great toe was observed (Figures 10, 11).

The children were followed up for the estimation of the LLD not exceeding 2 cm in total which was managed with a small shoe raise. Fillers were placed in front of the commercial shoe. In case of a further increase in LLD, treatment would be carried out with distraction osteogenesis.

A patient referred to us, with a clubfoot deformity (CTEV), had four rays on the affected foot. He was initially treated in another country in northern Europe. He had a discrepancy of 4 cm in both the femur and tibia. Equinus deformity of the foot was helpful at the age of 2 years to compensate for LLD. Hypoplasia of the lateral femoral condyle was also observed. The fibula was present on the affected side next to the proximal tibial epiphysis.

**Figure 12:**
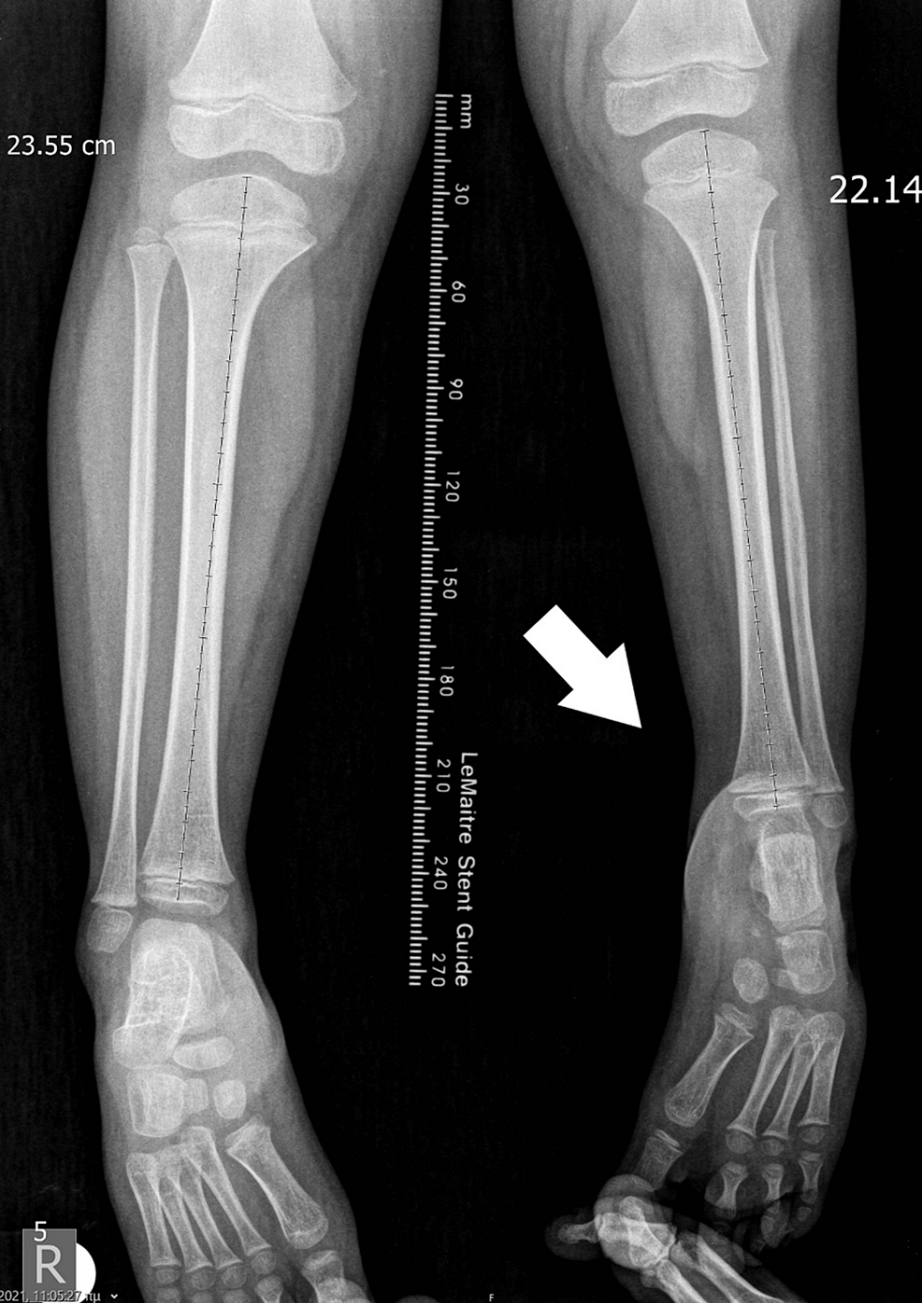
X-ray of the 4th patient with leg length discrepancy in the tibia.

**Figure 13:**
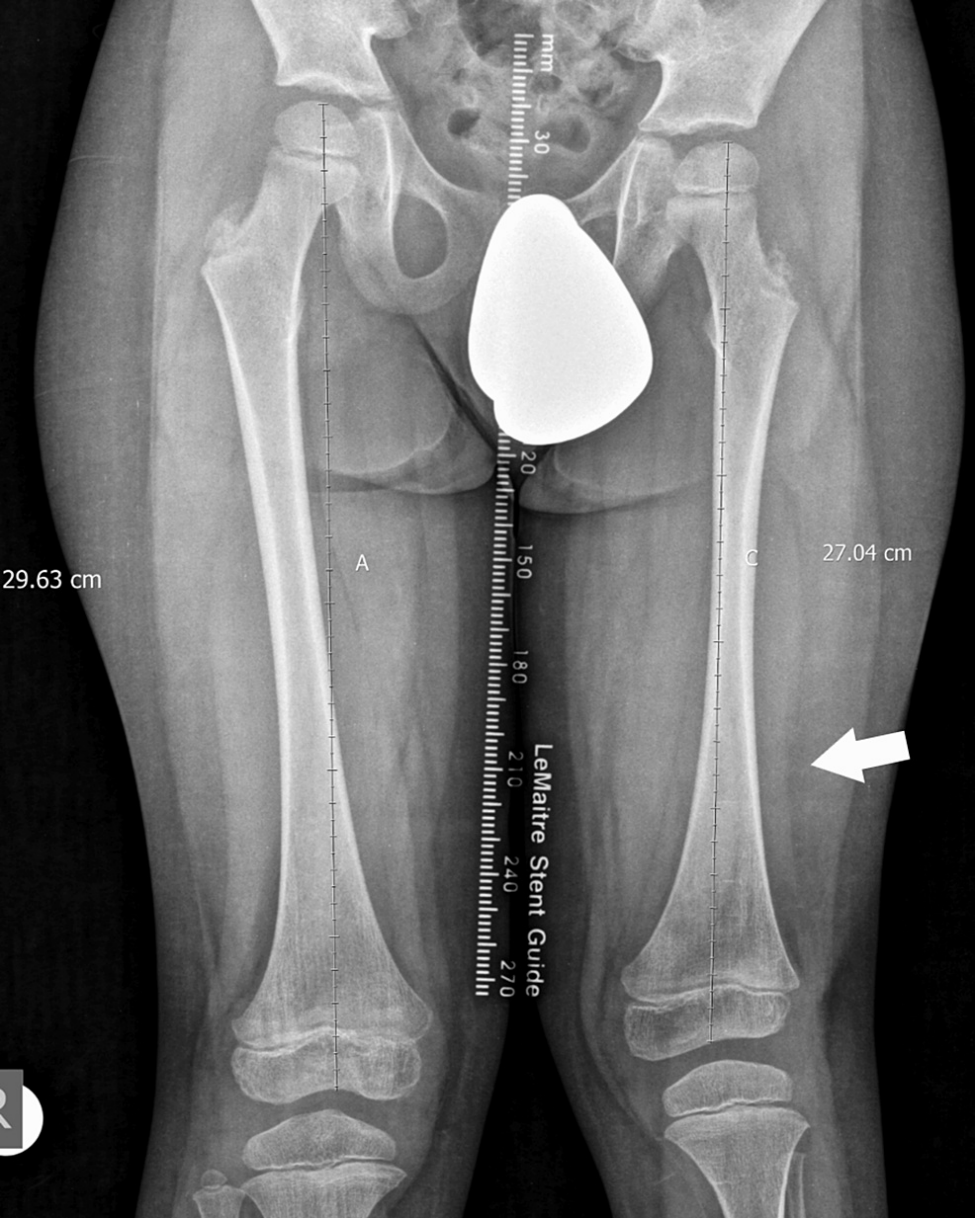
X-ray of the 4th patient, with leg length discrepancy in the femur.

**Figure 14:**
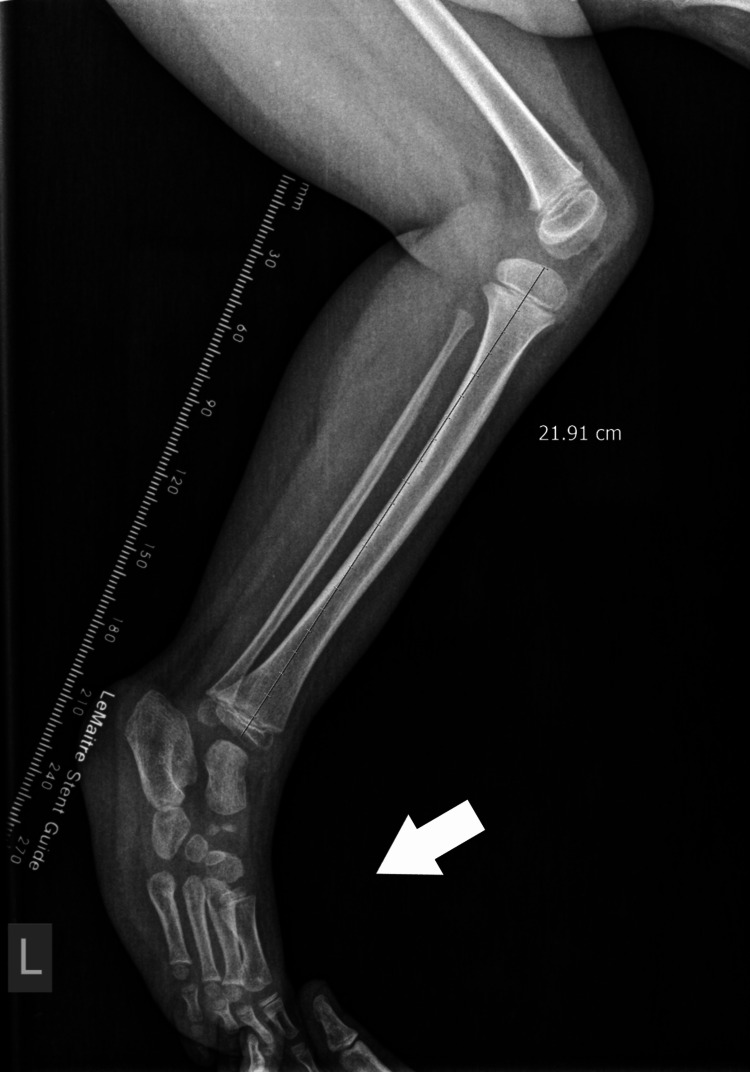
X-ray of the 4th patient with equinus foot, with delayed ossification of the navicular and normal cuboid bone.

The radiograph of the foot showed delayed ossification of the navicular associated with clubfoot deformity. The calcaneocuboid joint is present with two cuneiforms. As the child grows, we plan to perform distraction osteogenesis for the lengthening of the femur and tibia and correction of the equinus deformity (Figures 12-14).

**Figure 15:**
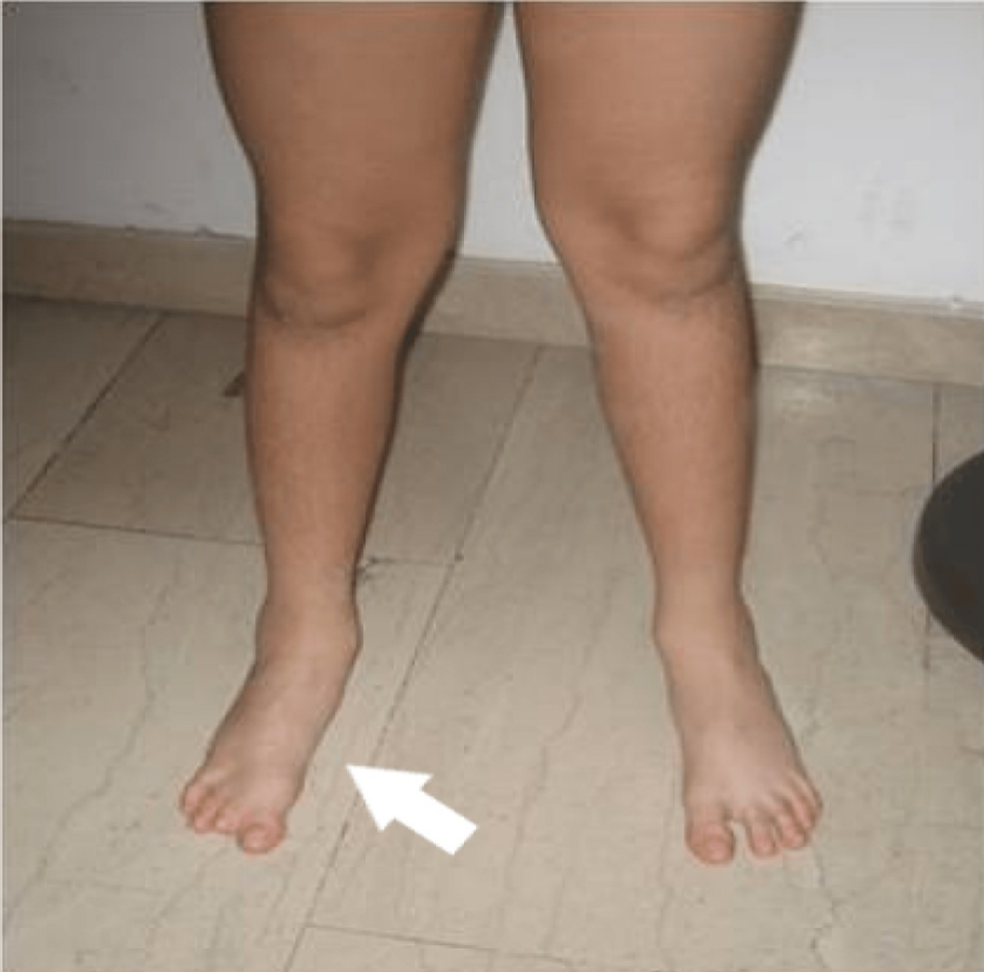
Clinical picture of the 5th patient with a plantigrade foot with minimal difference in size.

**Figure 16:**
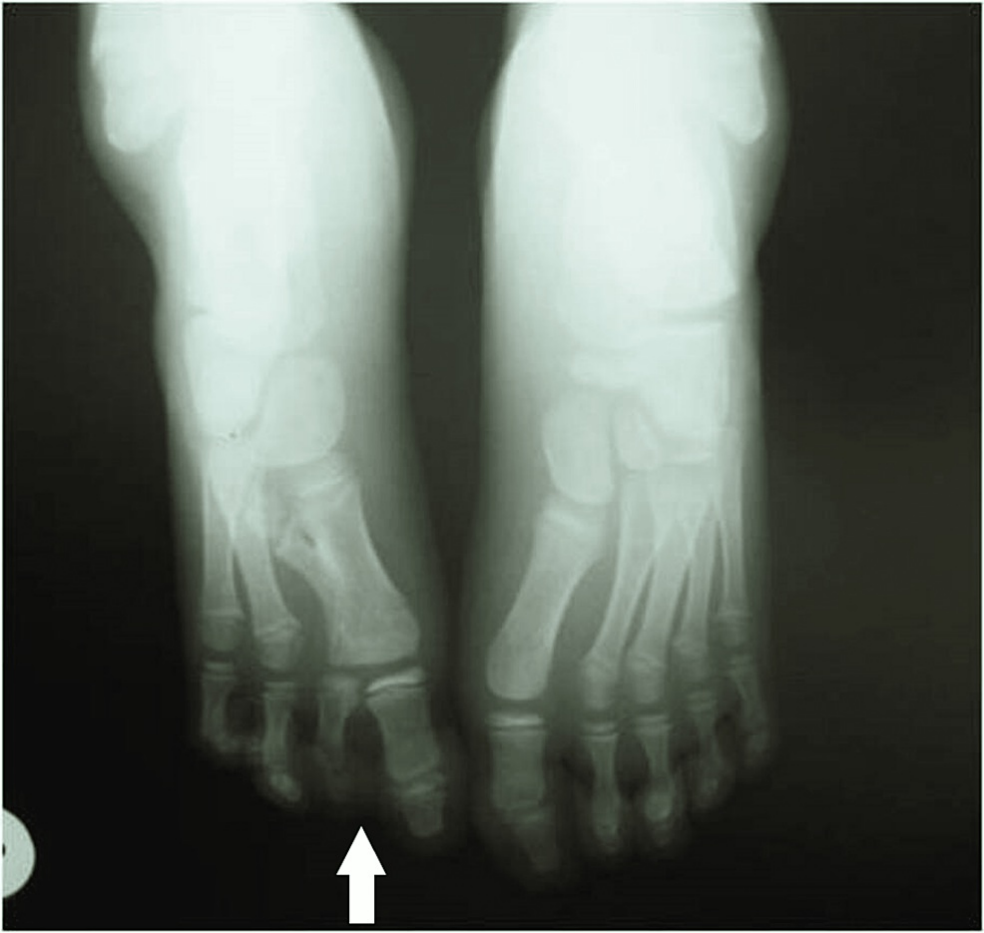
X-ray of the 5th patient with fusion of the hypoplastic 2nd metatarsal to the 1st one, absent navicular, normal calcaneocuboid joint.

**Figure 17:**
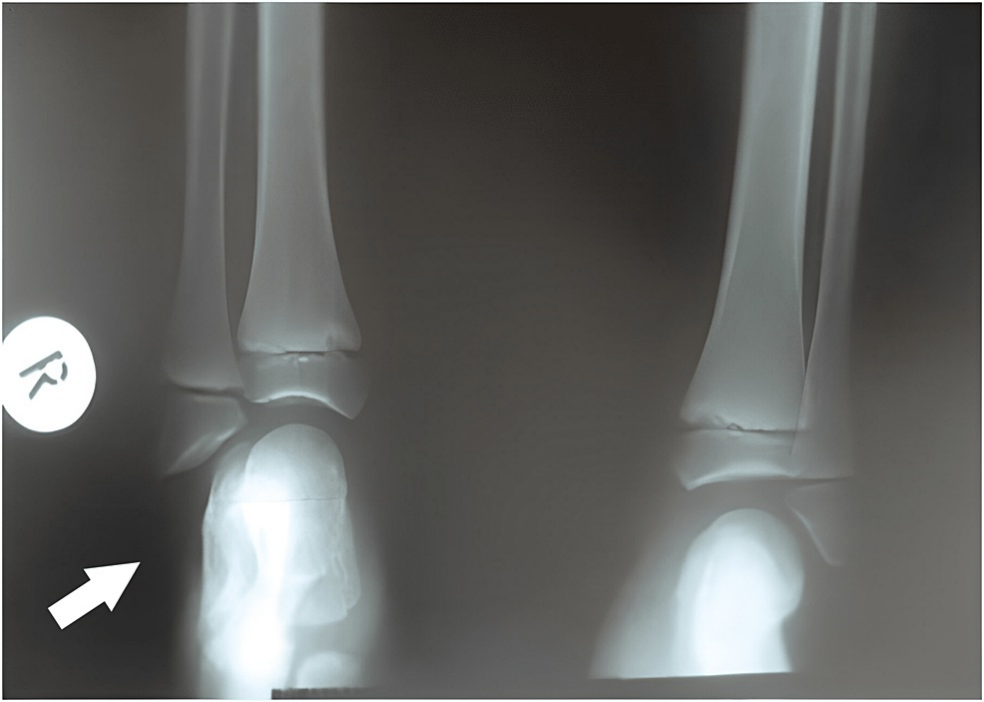
The ankle is a ball joint. X-ray of the 5th patient at the age of 5 years.

One boy had a complex deformity with an absent ray, fusion of the hypoplastic 2nd ray to 1st metatarsal, syndactyly of the hypoplastic 2nd toe, and an absence of the navicular. The ankle is a ball joint. A minimal LLD <1 cm was observed, and the calf diameter was slightly smaller. The boy participated in sporting activities, wearing normal shoes with fillers (Figures 15-17).

**Figure 18:**
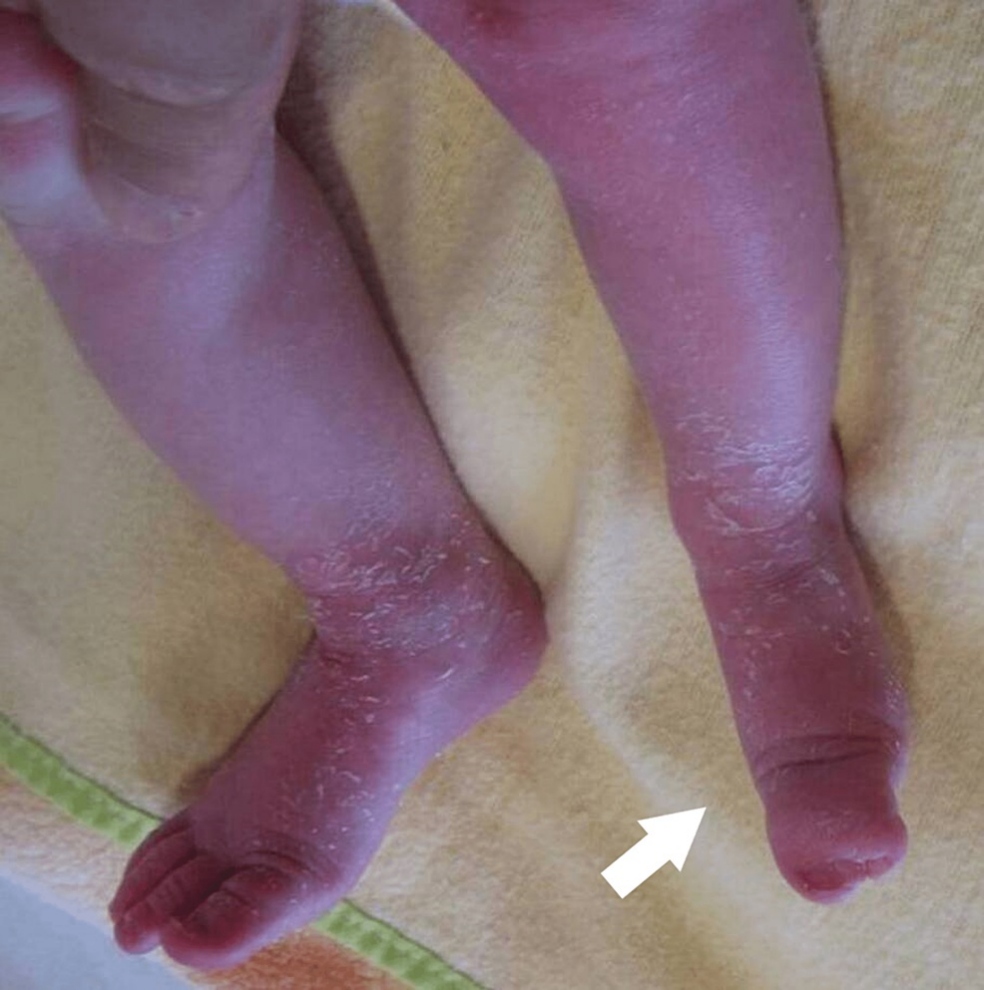
Clinical picture of the 6th patient's foot at the age of 6 months.

**Figure 19:**
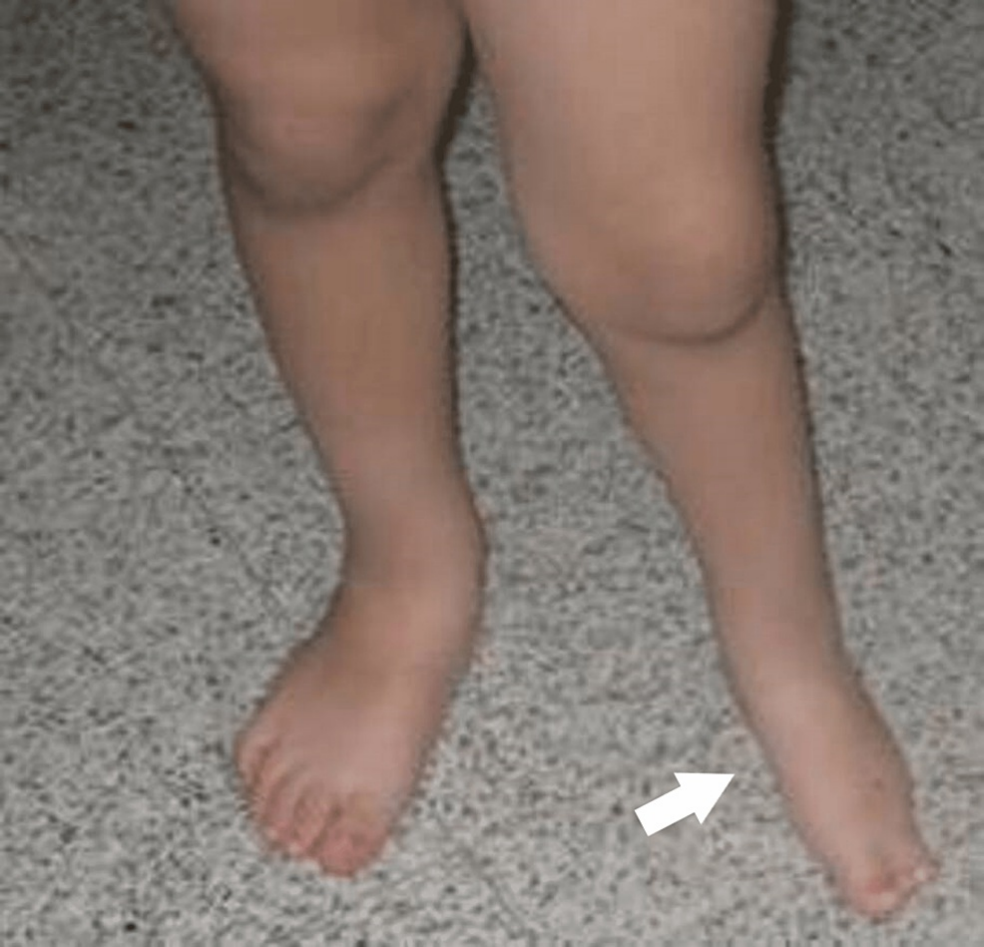
Clinical picture of the 6th patient's foot at the age of 3 years. There is a plantigrade foot, with LLD of 2 cm of the tibia. LLD: Leg length discrepancy

**Figure 20:**
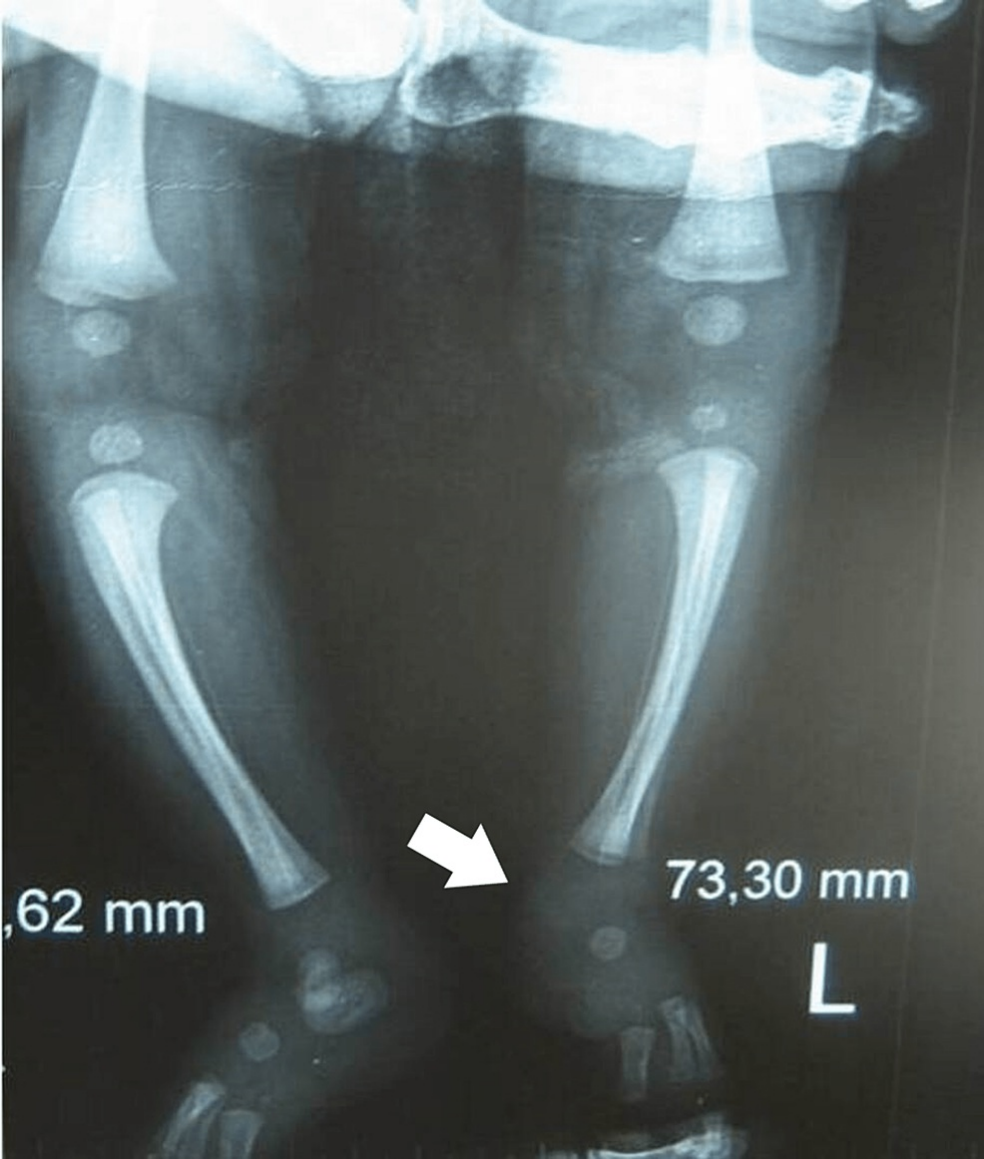
X-ray of the 6th patient at the age of 6 months. Initially delayed ossification of the heel, that later appears as a calcaneum-talus coalition with hypoplasia of the cuboid.

**Figure 21:**
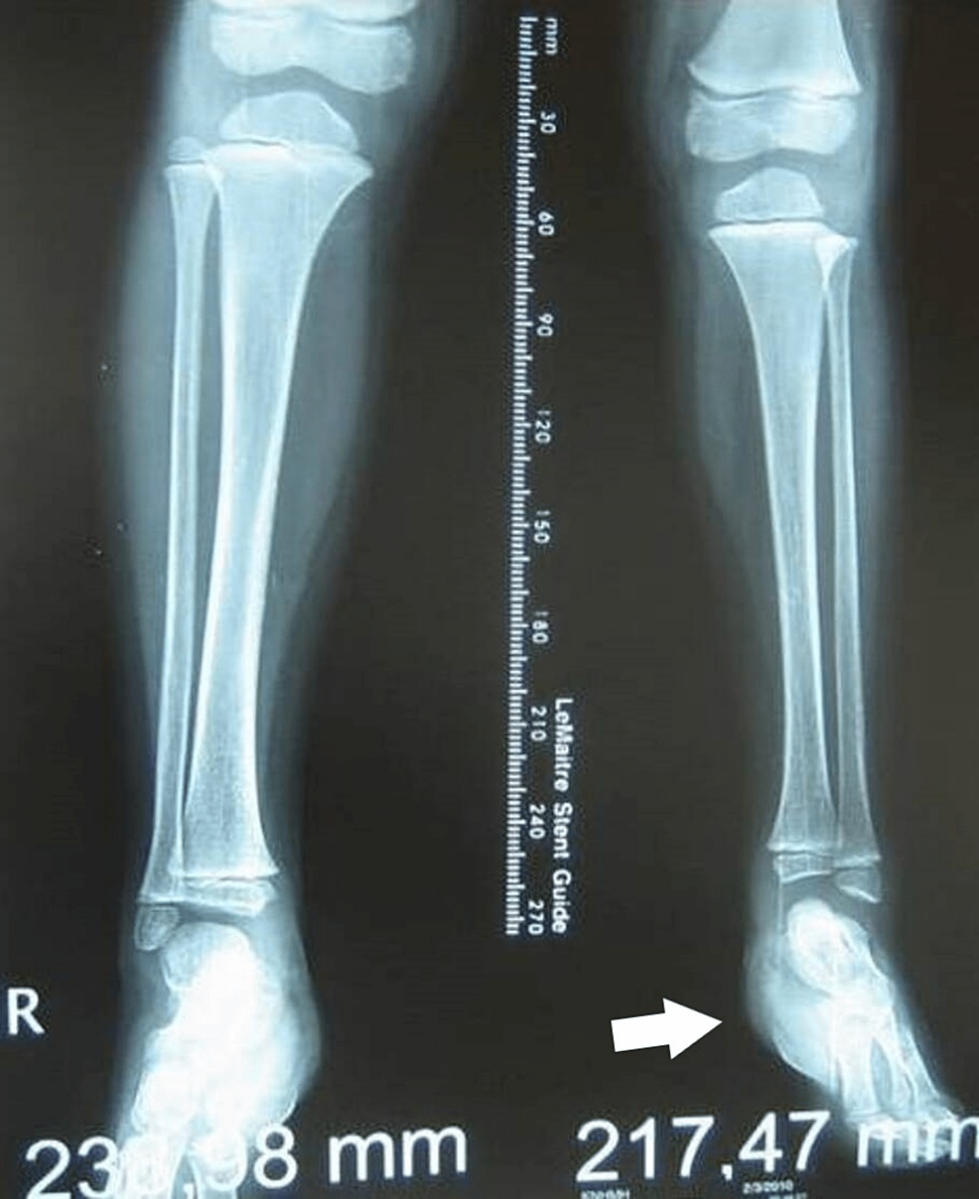
X-ray of the 6th patient at the age of 3 years. There is a ball joint ankle, with an LLD of 2 cm in the tibia. LLD: Leg length discrepancy

A girl with a unilateral three-ray foot, with hypoplastic toes and syndactyly had a coalition of the talus with the calcaneum, absent cuneiforms, and a hypoplastic cuboid. There was a ball-joint ankle. The leg length discrepancy at the age of 4 years was 2 cm. The girl had a plantigrade foot, participated in activities, and wore normal shoes with fillers. She was lost to follow-up at the age of 4 years (Figures 18-21).

The age, clinical evaluation, and radiological assessment of our oligodactyly patients appear in the following table (Table 1).

**Table 1:** Description of the patients.

Patient	Age	Rays	Foot Shape	Navicular	Number of cuneiforms	Calcaneocuboid	Coalition	Ankle joint	LLD
1st	7 years old	4 Bilateral	Normal	Present	2	Normal	Talo-Navicular Left	Normal	No
2nd	6 years old	4 Right	Normal	Hypoplastic	3	Normal	No	Ball Joint	1cm
3rd	7 years old	4 Right	Normal	Absent	2	Normal	No	Ball Joint	3cm
4th	3 years old	4 Left	Equinus	Hypoplastic	2	Normal	No	Normal	4cm
5th	5 years old	4 Right	Normal	Absent	2	Normal	Metatarsal 1-2	Ball Joint	1cm
6th	3 years old	3 Left	Normal	Absent	Unspecified	Unspecified	Hypoplastic Talus-Calcaneum	Ball Joint	2cm

## Discussion

A foot with a reduced number of rays must be properly evaluated as having isolated dysplasia or as part of hemimelia. We report oligodactyly with normal foot shape, as we did not include children with a cleft foot, which is commonly associated with reduced rays [4-7, 13-15].

Children with fibular deficiency present with a variety of limb formations and are classified according to Achterman and Kalamchi as type IA, IB, and II. In type IA, the fibula is hypoplastic but present, with the proximal and distal fibular epiphyses located distal to the proximal tibial epiphysis proximal to the dome of the talus. Reduced rays of the foot were described as absent lateral rays. Several concomitant features were found with tarsal coalitions, ball and joint ankle, valgus knee, and leg length discrepancy [1-3].

In a series with several patients over 72 years, 16 limbs of 14 patients were identified with signs of hemimelia without fibular hypoplasia. The authors reported that 13 of them had absent lateral rays and only three reported a five-ray foot. The foot shape was normal in 10 patients, and clubfoot deformity was found in four [16]. In our series, only one child had an equinus deformity, whereas the other five had a plantigrade foot including the child with the three-ray foot. A ball joint ankle joint, evident in four of our patients, presented without signs of instability.

Leg length discrepancy was a feature in all patients in our series, except the one with bilateral involvement. The discrepancy was not as severe as in the presence of fibular deficiency, which was the main problem in our patients with oligodactyly and type IB and II fibular deficiency. Leg length discrepancy greater than 3 cm was apparent in two of our patients who were scheduled to be treated with distraction osteogenesis. Similar small differences in LLD were described as an explanation for the smaller dysplasia of the whole limb [10, 11]. Oligodactyly, as a localized dysplasia, appears without the severe LLD as in cases of fibular absence or severe deficiency [17].

Our group has some distinct radiological features that have not been previously described. The absence or decreased size of the navicular was a radiological finding in five children in our group.

Based on the presence of a normal calcaneocuboid joint, the term intermediate ray deficiency was used to describe feet with oligodactyly and normal fibula [10]. The authors reported on a series of nine patients with a normal calcaneocuboid joint. They mainly focused on the elements of hypoplasia, with emphasis on the LLD, ball joint ankle, and valgus knee deviation. They described that in eight of the nine patients, there were two cuneiforms and only one child had three cuneiforms. In our children with two cuneiforms, it was difficult to clinically distinguish which ray was absent. The absence of the navicular suggested a lesion of the 1st ray but the shape and length of the medial 1st ray were normal. Delayed ossification of the navicular and smaller size may be found in CTEV, as was noticed in two children; one with a history of clubfoot deformity. They also described the shape of the foot as hypoplastic with a normal shape of the medial and lateral rays [10]. This is the same as the shape of the foot of our children including the girl with the three-ray foot who had a plantigrade foot, and the shoewear was normal with slight modification.

Baek et al. compared the leg length discrepancy between the tibia and fibula on the defective and normal sides in two groups of children with oligodactyly [11]. They used the term terminal hemimelia group when the LLD of the tibiae was the same as that of the fibula between the two sides. There were five children in this group, two of which had a three-ray foot. They found a stable ankle joint with a ball joint shape in all cases and a tarsal coalition in four of them. They described the absent lateral ray, but in the radiograph of the foot in their paper, the navicular was also missing, as well as the cuboid. They also reported an expected LLD of 2.65 cm at maturity, similar to our patients. The patient did not require foot procedures. They were surgically treated for leg length discrepancies.

A radiological review of foot ray deficiency in all children with fibular deficiency was conducted to clarify the terminology of lateral or central ray deficiency [18-21]. Twenty-five children were included in the study for all types of fibular deficiencies. The lateral ray was reported to be normal with the presence of a well-developed cuboid or calcaneocuboid coalition, with which the most lateral metatarsal was articulating. This was observed in all 25 children. In their series, six feet had three rays and two cuneiforms. They described 12 feet with four rays and two cuneiforms. In only two of them, they described a separate navicular and the remaining had a talonavicular coalition (eight feet). In two feet, the navicular was uncharacterized [18]. Our group of patients refers to children with normal fibulae, with the same characteristics as those without a navicular or talonavicular coalition. Their group also had children with the plantigrade foot, and in three of them, there was an equinovarus deformity. None of the patients had a planovalgus deformity. The authors favored a deficiency of the midline metatarsals [18-21].

Prenatal diagnosis of the absent rays created major anxiety in the parents of the fetus, which could be part of a major dysplasia or a syndrome. In a recent report, bilateral split-hand and foot deformities were detected prenatally. The father had bilateral oligodactyly with syndactyly, and elective termination of pregnancy was decided [7]. In two of our children, there was a prenatal diagnosis of oligodactyly. With normal measurements of the limbs, the absence of other dysplasia, and the presence of fibulae, parents were informed of the course of isolated dysplasia.

The main characteristic of our group of patients was the absence of a foot ray, with a normal or minimally affected fibula. We separated this from children with type IB and type II fibular hemimelia. Our children had a normal shape of a plantigrade foot, apart from one with equinus deformity. A leg length discrepancy exceeding 3 cm was found in two of them. They require a leg-length equalization procedure. There were a variety of bone deficiencies affecting both the medial and central parts of the foot, with hypoplastic or absent navicular and cuneiforms, and further complex synostoses. We must emphasize that oligodactyly may be an isolated dysplasia, with particular attention to the management of LLD.

As for the limitations of our study, we have evaluated the feet with plain X-rays, and possibly the use of a CT scan could have revealed more malformations and coalitions affecting the bones. Follow-up of children until the end of the growth is required to evaluate appropriately the final LLD.

## Conclusions

A cohort of six children with a reduced number of rays of the foot is reported. Oligodactyly was an isolated dysplasia and radiological examination featured the missing or hypoplastic bones of the foot. Leg length discrepancy was the main problem that must be treated in later life. LLD in our patients was smaller compared with the discrepancy in oligodactyly as part of fibular or tibial insufficiency. Appropriate prenatal counseling for the isolated oligodactyly is important.
